# Supplementary material for: A novel form of JARID2 is required for differentiation in lineage‐committed cells
Source: EMBO J. 2018 Dec 20;38(3):e98449. doi: 10.15252/embj.201798449 (PMC6356158; doi:10.15252/embj.201798449)
Supplement: Supplementary file 1 — Appendix [file EMBJ-38-e98449-s001.pdf]

Table of Contents

Appendix Methods 2
- Cell culture and differentiation 2
- Transfections 2
- CRISPR/Cas9-mediated genome editing 3
- qRT-PCR 4
- Histone extraction 4
- Immunoblotting 5
- Immunofluorescence 5
- References: 5

Appendix data 7
- Sequences for RT-PCR product E3-E15 from Fig 2D 7

Appendix Tables 8
- Table S1. siRNAs sequences used in this study 8
- Table S2. Primers used in this study for qPCR 8
- Table S3. Primers used to amplify JARID2 9

Appendix Figures 10
- Figure S1. Expression of ΔN-JARID2 from an exogenous vector 10
- Figure S2. Analysis of functional categories in genes affected by JARID2 knockout 11
- Figure S3. Interaction bewteen JARID2 and polycomb protein EZH2. 12
- Figure S4. Interaction of JARID2 with AP-1 13

Appendix Methods

Cell culture and differentiation

HaCaT, a spontaneously immortalized human keratinocytes cell line was grown in Dulbecco's modified eagle medium (DMEM) including high glucose, calcium and glutamine containing 5% Fetal bovine serum (FBS) and 1% penicillin-streptomycin (10.000U/mL) in 5% humidified CO_2 incubator at 37°C. Cells were trypsinized when they had reached ~80% confluence using 0.05% Trypsin-EDTA. To maintain HaCaT cells in basal or undifferentiated phenotype as previously described (Wilson, 2014), 80% confluent HaCaT cells were cultured in calcium-free DMEM containing high glucose, no glutamine and no calcium with 10% chelated FBS, 200mM L-Glutamine in addition to 0.03mM calcium chloride for four weeks at 80% confluence. FBS was incubated with Chelex 100 resin (BioRad#143-2832) for 1h at 4°C to remove calcium from FBS and then was filtered using the Stericup filter unit (Millipore; 0.22um pore). For calcium-induced differentiation, 80% confluent basal/undifferentiated HaCaT cells were trypsinized using 0.05% Trypsin-EDTA (life technologies). HaCaT cells were seeded in 10cm^2 dishes and incubated with low-calcium (0.03mM) growth medium overnight to reattach the cells at 80% for control and full confluence (100%) for the rest of dishes. The medium was replaced with high calcium (2.8mM) growth medium to induce the cells to differentiate at different time points. The medium was refreshed every two days. For EZH2 inhibition, cells were grown overnight and treated with 3µM of UNC1999 for 24h then collected for immunoblotting (Xu et al., 2015).

Transfections

Reverse or transient knockdown of JARID2 was done using Lipofectamine RNAiMAX (Invitrogen). Following manufacturer's instructions, in 6-well plate 500µl of reduced serum medium (Opti-MEM, Invitrogen) was mixed with 8µl (80pmol) of siRNAs and incubate for 10mins and 5µl of lipofectamine RNAiMAX reagent was then added. After mixing, the mixture was incubated for 10min. Approximately 2X10^5 HaCaT cells were added into mixture with 2ml of growth medium. Cells were incubated for 48h before harvesting. Non-silencing control (NSC) was used at the same concentration of siRNAs.

To transfect HaCaT cells with DNA, Amaxa Cell Line Nucleofector kit V (Lonza) was

used. Following manufacturer's instructions, HaCaT cells (3 x10^6) were resuspended with 100µl nucleofector solution. Cells were then electroporated with 5-10µg DNA using nucleofector device. U-20 program was optimized for HaCaT cells as recommended by manufacture. The mixture was plated in 6 wells plate containing pre-warmed medium and incubated for 24h. The medium was changed after 24h with fresh medium. To generate stable cell lines, cells were grown in DMEM medium containing 10µg/ml of Blasticidin (Heaton et al., 2011) or 500µg/ml G418 (Wang et al., 2014) antibiotics.

For HEK-293T transfection, in 6 wells plate cells (1 x10^5) were grown in DMEM supplemented with 5% FBS overnight. DNA (1-2µg) was mixed with 100-200µl reduced serum medium (Opti-MEM). X-tremeGENE9 DNA transfection reagent (Roche) was added as 3:1 ratio of reagent to DNA. The mixture was incubated for 15min at RT. Finally, transfection complex was added to cells in a dropwise manner and incubated for 48h before proceeding with immunoblotting.

CRISPR/Cas9-mediated genome editing

Single guide RNA (sgRNA) for JARID2 was designed using online tool, Wellcome Trust Sanger Institute Genome Editing database (WGE) (Hodgkins et al., 2015). JARID2 sgRNAs consist of 20nt and NGG sequence; protospacer adjacent motif (PAM) on forward or positive strand (Fig EV2A). sgRNA sequences were selected with minimum off-target effects and close to translational start site (ATG). Following design guides, CACC was added before guide sequence and AAAC was added in reverse complementary guide sequence. In case if the first nucleotide of guide sequence is not G, it was added after CACC and C nucleotide was added at the 3' end of reverse complementary sequence. Primers of sgRNA were ordered from sigma without including PAM sequences (Bauer et al., 2015). Forward and reverse primers (100µM) were mixed with 10x annealing buffer (100mM NaCl and 5mM HEPES pH 7.4) at 90°C for 4min, 70°C for 10min and 10 ^{o}C for 10 min. pX459 was digested with BpiI (TheremoFisher Scientific) at 37°C for 3h. Annealed oligonucleotides were ligated with 10-50ng of pX459 plasmid using T4 DNA ligase (New England Biolabs). The reaction was incubated overnight at 16 ^{o}C and then was transformed to competent DH10B *E.coli* cells, plated onto agar plates containing ampicillin (100µg/ml) and grown at 37°C overnight. DNA was extracted and sequenced using U6 promoter primer. 5µg of DNA was transfected to HaCaT cells

using electroporation and cells incubated for 24h. Transfected cells were selected for 3 days in medium containing 0.5µg/ml puromycin (Ryan et al., 2012). Following selection, cells were maintained in low calcium medium for 5-7 days. Using single cell dilution, $1X10^4$ cells/ml were serially diluted until 0.5cell/100µl. In flat bottom 96 wells plate, 100µl was added in each well (i.e. one cell per two wells). After 4 days, the plate was visually screened to select only wells containing single colony. Single cell was grown in low calcium medium.

DNA was isolated from single clone JARID2 CRISPR knockout and wild-type HaCaT cells using PureLink Genomic DNA Mini Kit (Invitrogen). Amplification of targeted loci was done using Q5 High-Fidelity DNA polymerase (New England Biolabs) and specific primers on JARID2 gene (F: GTGATTCTTTTACTTAAGTGGCCAATG and R: CTAGACCAGGAAGCACAAGCTG). PCR products of successfully mutated cells were cloned into pJET1.2 blunt vector (ThermoFisher Scientific). 50ng of purified PCR products was ligated into pJET1.2 blunt vector (3:1 molar ratio of insert to vector) using T4 DNA ligase and incubated for 5-20min. 5µl of ligation reaction was transformed into competent DH10B *E.coli* cells and plated onto agar plates containing ampicillin. At least 10 bacterial colonies were picked up for sequencing to identify mutations.

qRT-PCR

Total RNA was extracted from cultured cells using RNeasy Mini Kit (Qiagen, Germany), treated with Amplification Grade DNase I (sigma) to remove genomic DNA and quantified by NanoDrop. cDNA was synthesized using Tetro cDNA synthesis kit (Bioline). RT-PCR was performed in triplicate using SensiFAST SYBR Hi-ROX kit (Bioline) and Agilent AriaMx (Agilent Technology). Samples were normalized to 18S rRNA or GAPDH using $\Delta\Delta$CT method. Primer sequences used for RT-PCR are available in supplementary (Table S2).

Histone extraction

Histone extraction was done in collaboration with Dr. John Halsall (Chromatin and Gene Expression Group, College of Medical and Dental Sciences). Cell pellets at ~$1x10^7$ cells/ml were lysed in triton extraction buffer (0.5% Triton x100, 2mM PMSF, 0.02% Sodium Azide in 1X PBS) for 1 minutes on ice and followed by centrifugation at 500g for 10 minutes at 4°C. After discarding the supernatant, the pellet was washed in half the original volume of triton extraction buffer and then resuspended in

0.4M hydrochloric acid. Histones were extracted at 4°C overnight, centrifuged and the supernatant was subjected to western blot after measuring protein concentration using Pierce Coomassie Plus Bradford Assay Reagent (life technologies).

Immunoblotting

The samples were treated with 4x sample buffer (200mM Tris HCL pH 6.8, 40% glycerol, 4% SDS, 0.4% bromophenol blue and 200mM β-mercaptoethanol) and then heated at 70°C for 10 min. Total protein lysates were loaded, separated by 12-10% SDS-PAGE. The blots were transferred onto nitrocellulose membrane (BioRad) using Trans-Blot Turbo Transfer System (BioRad). The blots were then blocked with 5% skimmed milk in Tris Buffered Saline (TBS) for 1h. After blocking, the membranes were incubated with primary antibody at 4°C in the shaker overnight. The blots were washed three times for 5 min with TBS- 0.05% Tween 20, pH 7.5. Then, blots were incubated for 1h with fluorescent goat anti-rabbit or anti-mouse secondary antibodies (1:15000, IRDye, LI-COR) diluted in 5% skimmed milk. They were washed three times for 5 min with TBST. The blots were scanned using odyssey infrared detection system (LI-COR Biosciences). Bands were quantified using Image Studio Lite (LI-COR Biosciences) and normalized to GAPDH.

Immunofluorescence

Cells were cultured on autoclaved and treated cover slips with pure nitric acid at room temperature, washed three times with PBS and then fixed in PBS containing 4% (w/v) paraformaldehyde for 10min at room temperature. Following this, cells were permeabilised with 0.2% Triton X100 in PBS for 3min on ice. After three washing with PBS, cells were blocked with 5% BSA for 1h and incubated with primary antibody overnight. Following incubation with primary antibody, cells were washed three times with PBS and were then incubated with Fluorescein (FITC)-AffiniPure donkey anti-Mouse IgG secondary antibody (Jackson ImmunoResearch Laboratories Inc) (1:600) for 1h. After washing three times with PBS and once with water, cells were finally mounted with vectashield with DAPI and analysed by Nikon A1R confocal microscope (Nikon, UK ltd).

References:

Bauer, D.E., Canver, M.C. and Orkin, S.H. (2015) Generation of genomic deletions in mammalian cell lines via CRISPR/Cas9. J Vis Exp*,* (95): e52118.

Heaton, P.R., Deyrieux, A.F., Bian, X.L., et al. (2011) HPV E6 proteins target Ubc9, the SUMO conjugating enzyme. Virus Res*,* 158: (1-2): 199-208.

Hodgkins, A., Farne, A., Perera, S., et al. (2015) WGE: a CRISPR database for genome engineering. Bioinformatics, 31: (18): 3078-3080.
Ryan, K.R., Lock, F.E., Heath, J.K., et al. (2012) Plakoglobin-dependent regulation of keratinocyte apoptosis by Rnd3. J Cell Sci, 125: (Pt 13): 3202-3209.
Wang, Y., Li, X., Song, S., et al. (2014) Development of Basal-Like HaCaT Keratinocytes Containing the Genome of Human Papillomavirus (HPV) Type 11 for Screening of Anti-HPV Effects. J Biomol Screen, 19: (8): 1154-1163.
Wilson, V.G. (2014) Growth and differentiation of HaCaT keratinocytes. Methods Mol Biol, 1195: 33-41.
Xu, B., On, D.M., Ma, A., et al. (2015) Selective inhibition of EZH2 and EZH1 enzymatic activity by a small molecule suppresses MLL-rearranged leukemia. Blood, 125: (2): 346-357.

Appendix data

Sequences for RT-PCR product E3-E15 from Fig 2D

>E3-E15_sequence_part1

```
ATRARMMYMWTGTACGGGAGTCCTCGAATGTCATCTCTGGGTGCAGGTGTAACCAGTGCCAA
AAAGATGCGCGAGGTCAGACYTTCACCATCCAAAACTGTGAAGTACACTGCCACGGTGACGA
AGGGGGCTGTCACATACACCAAAGCCAAGAGAGAACTGGTCAAGGACACCAAACCCAATCAC
CACAAGCCCAGTTCCGCTGTCAACCACACAATCTCAGGGAAAACTGAAAGTAGCAATGCAAA
AACCCGCAAACAGGTGCTATCCCTCGGGGGGGCGTCCAAGTCCACTGGGCCCGCCGTCAATG
GCCTCAAGGTCAGTGGCAGGTTGAACCCAAAGTCATGCACTAAGGAGGTGGGGGGGCGGCAG
CTGCGGGAGGGCCTGCARCTGCGGGAGGGGCTGCGGAACTCCAAGAGGAGACTGGAAGAGGC
ACACCARGCGGAGAAGCCGCAGTCGCCCCCCATGAWSATGAAAGGGGCGGCTGGTCCCTCCG
AAKGYCCTGRCAAGATGGYCCCGGCCCATATTTGTCTGCTGTTTGGATGCGTGARTAATTCY
TG
```

>E3-E15_sequence_part2

```
AWAAAACARACTMTTKCTCMTYGGTGGTAGCAAGTGCACGGTTTCAGACACGCTGTACCCAC
AGCACACTTTGGACACAAAGGATCCCGGGAAGCAGACGACAAACTGGCCACTCTGCTGCACG
GTCCTGTGCACCTTGATCCCCTCTTTGCACAGCACCTCCGGGGAGATCATGACGTTGCTTTC
CAGCATCTGCAGCCCTGGGGTGCCATTGGCTTGCAGCAGGGTGTGGACCACATCTTCCAGCT
TGTTCTCCTCCTCAGCAGGAATGCAATACCAAATGCAGTCAGCACCAGTGTGTAAGTAGTCA
ATGTATGGAAGGTGATTTTGGTCTCGAGACCAGCATGAGGTAGAAAAGACCATGCCAATATT
TAGCCAGGGAATAGTCACTCCAGGCACAGCACCGAGGTGACGCAGGATGGACCCTGTGTTAT
TGGGGAGGACGGTGAGGTTCCATCCATGCCTCGAAAAGGGTTCTGATTTTCCTACTGGGAAT
CCACTGCCGTGAGTGTTGGTGTCCACCTTGCCGCAGTGCACTGCCACGTGGCAGTCCTTCTC
TTCCACTAGCCTCCAGTACTCTTGCTCGATTTCGGCTGGGGCAGGCTCCTTGCTGAAACACA
TGCTCATGATATTCCTCGCTGTTCGATAAAAAGTTGTTAGAGAAACAGACCTTCCCTTATAG
ATGCACTTGTGGAAGTCATTGAGGACGCCTTTGTCCTCCTCCTCTTCCTTGACCACTTCTTT
TTCCTGAGCGAAGAGTCGCCGCCGGCCAGTCTTCAACTGGGCCTGGCCCACCTCCTTGAGCT
TGCTGCGGAAGCCGTTCCTGGGGGCCACGCCGTGGATGAGCCCATTCTTGGGCTCGAAGCGG
GGCAGAGGGTGGAACTTGTGGTGGTCGTTCTCTGTGTGGCCTTCCAGCGGCCCCTTGCGCTT
CTCCAGGATCTCCTTCTCCATCAGCACCTCCTTCTCCAGCCGCCGGTGCTCCTCTGGGGAMA
GGGAGTCGTAGAGAGWAGWAMTGGCAGTAAGCYTCCTGCARCTTGGCCAGCCGGTCCTGGGC
AGTTCTGGGATGCKCAACAWGTCTGCTARTTGTTCCATTTTTTGAAGGTCAGTCAYTTGCTG
CATGCGCCATTCTCATAATCAGCCGTAAAGCAGGCATGYGAGTCTCAGTCCCCTWTGACGGA
GCTYGTCATTKGKAWGGCCTGKGCATTGAAKTCTCTTGATGCAGGCAACCGGCKACGTTGGR
GACCCAASAGCTGCARCTTGGTATGAAKWGTGCTG
```

Appendix Tables

Table S1. siRNAs sequences used in this study

Oligos	Sequence 5'-3'
JARID2 siRNA1	GGGAUUAGGACCAGCAUCA
JARID2 siRNA2	GCCAUAUAGCUAAGCCAUU

Table S2. Primers used in this study for qPCR

Gene	Sequence 5'-3'
JARID2 isoform-1F	GTCCCCTTTTGCAATCAGCA
JARID2 isoform-1R	TCCCATCACTGTCATCGTATTTCT
JARID2 isoform-2F	GCTTCCCACCAGGATGACAG
JARID2 isoform-2R	CTTCCGCATGCTGCCTCTTC
JARID2 isoform-3F	GGGTCTGTCAGGTGCAGTTT
JARID2 isoform-3R	TGACTGTTCTGATGCTGGTCC
IVL F	CGCCATCCAGGAAAAGGTGT
IVL R	AAGCGCTTGGTGGTCAGATT
KRT1F	GTTCCAGCGTGAAGTTTGTT
KRT1R	TAAGGCTGGGACAAATCGAC
KRT10F	GCAAATTGAGAGCCTGACTG
KRT10R	CAGTGGACACA TTTCGAAGG
18SrRNA F	GCAATTATTCCCCATGAACG
18SrRNA R	GGCCTCACTAAACCATCCAA

Table S3. Primers used to amplify JARID2

Gene	Sequence 5’-3’
JARID2 (Exon1) F	AGCAAGGAAAGACCCAAGAGGAATATCA
JARID2 (Exon18) R	CATGATGAGCTCGAAGCACTTTTGG
JARID2 (Exon3) F	GGGATTAGGACCAGCATCAGAAC
JARID2 (Exon15) R	AATGGCTTAGCTATATGGCGACG

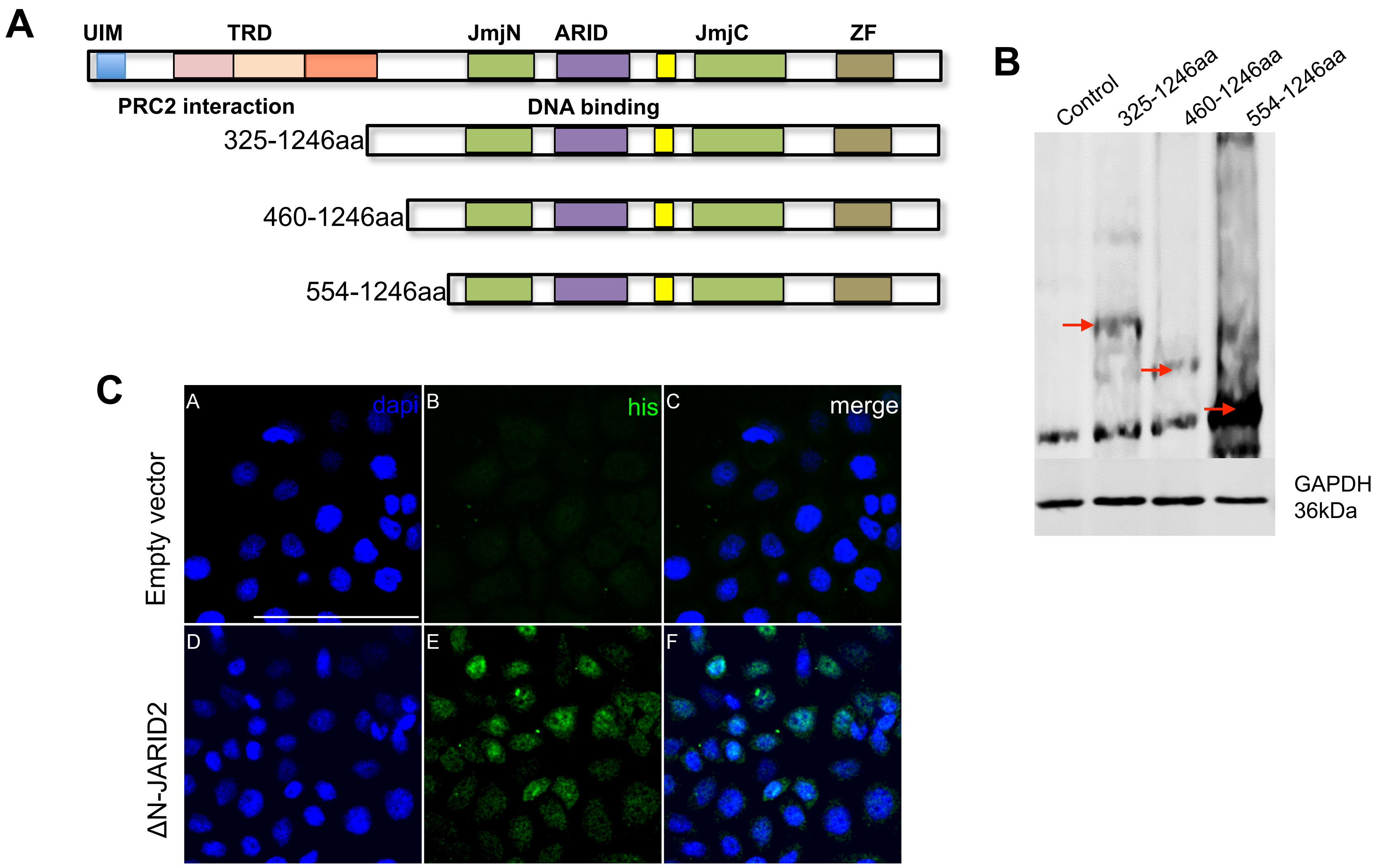

Figure S1. Expression of ΔN-JARID2 from an exogenous vector A) Different C-terminal fragments of JARID2 were cloned and exogenously expressed using a His-tag vector. **B**) Immunoblotting of protein extracts from transfected HEK-293T cells with anti-JARID2 antibody to confirm the size of the JARID2 LMW form relative to the cloned fragments shown in A. **C)** Immunostaining of HaCaT cells expressing the fragment (554-1246aa) corresponding to ΔN-JARID2. Cells were stained with DAPI (blue) and anti-His antibody (green)

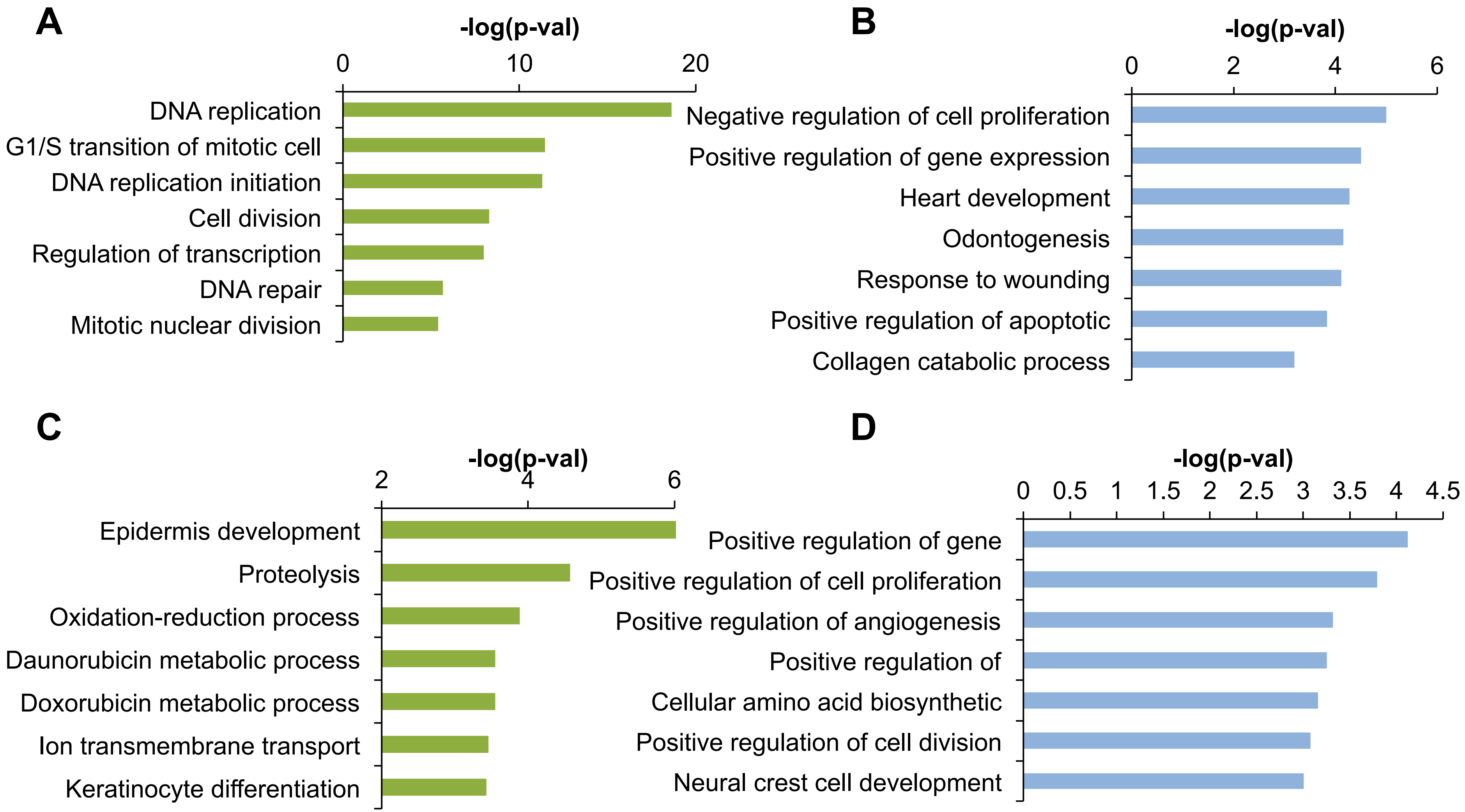

Figure S2. Analysis of functional categories in genes affected by JARID2 knockout.
A) Functional categories of genes which are 2-fold or more up-regulated and (B) down-regulated in JARID2-null cells grown in low calcium. Functional categories of genes that are 2-fold (C) up-regulated and (D) down-regulated when wild-type cells are differentiated in high-calcium medium over a period of 3 days.

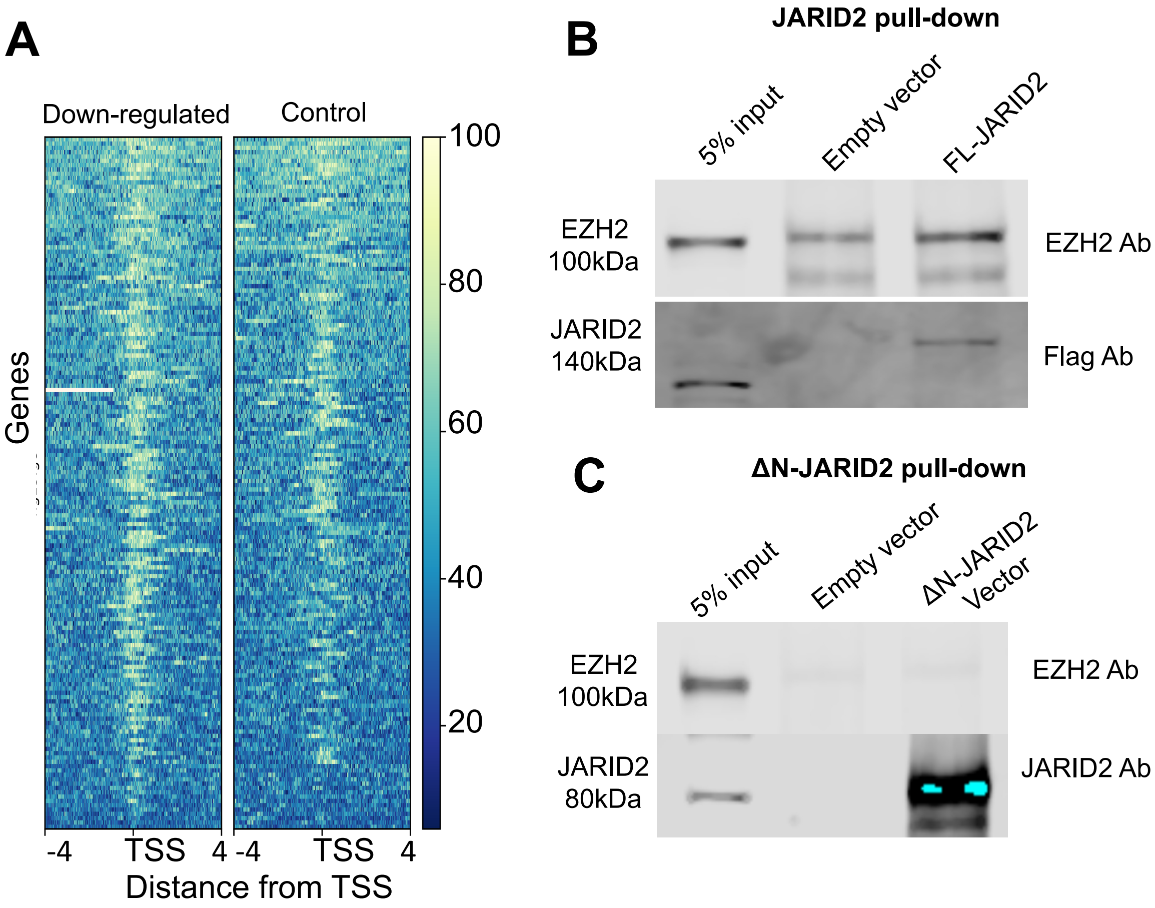

Figure S3. Interaction bewteen JARID2 and polycomb protein EZH2.
A) Heatmaps showing GC distribution at down-regulated vs randomly selected control set of genes. B) Immunoblot showing that EZH2 is co-immunoprecipitated with full-length JARID2. **C)** Immunoblot confirming the lack of interaction between EZH2 with ΔN-JARID2. EZH2 cannot be co-immunoprecipitated with ΔN-JARID2.

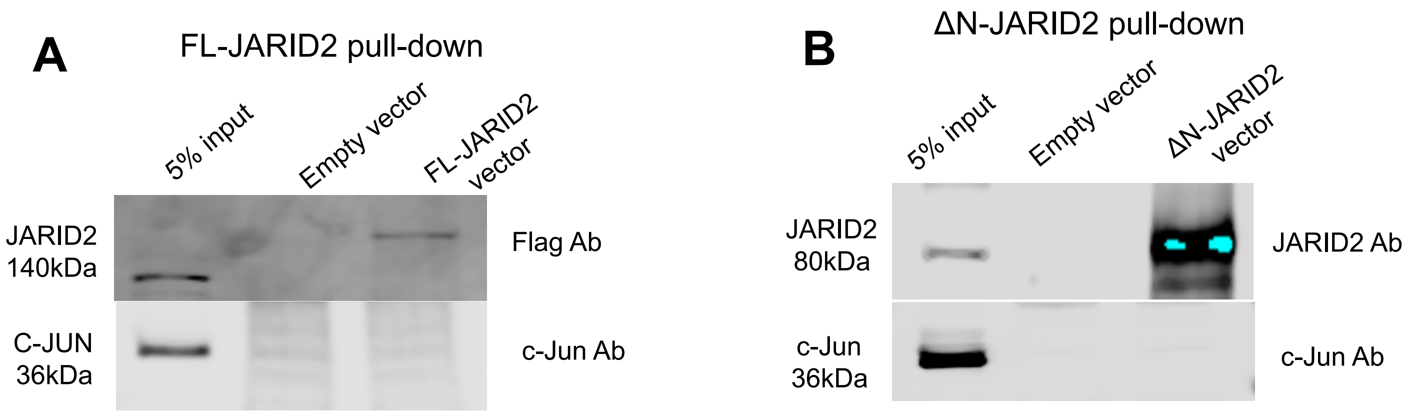

Figure S4. Interaction of JARID2 with AP-1

A) Immunoblot showing that the AP-1 protein c-Jun is not co-immunoprecipitated with full-length JARID2 (FL-JARID2). The blot from Figure S3B was re-blotted with c-Jun antibody **B)** Immunoblot showing c-Jun does not co-immunoprecipitate with ΔN-JARID2. The blot from Figure S3C was re-blotted with c-Jun antibody
