# Supplementary material for: A novel form of JARID2 is required for differentiation in lineage‐committed cells
Source: EMBO J. 2018 Dec 20;38(3):e98449. doi: 10.15252/embj.201798449 (PMC6356158; doi:10.15252/embj.201798449)
Supplement: Supplementary file 2 — Expanded View Figures PDF [file EMBJ-38-e98449-s002.pdf]

Expanded View Figures

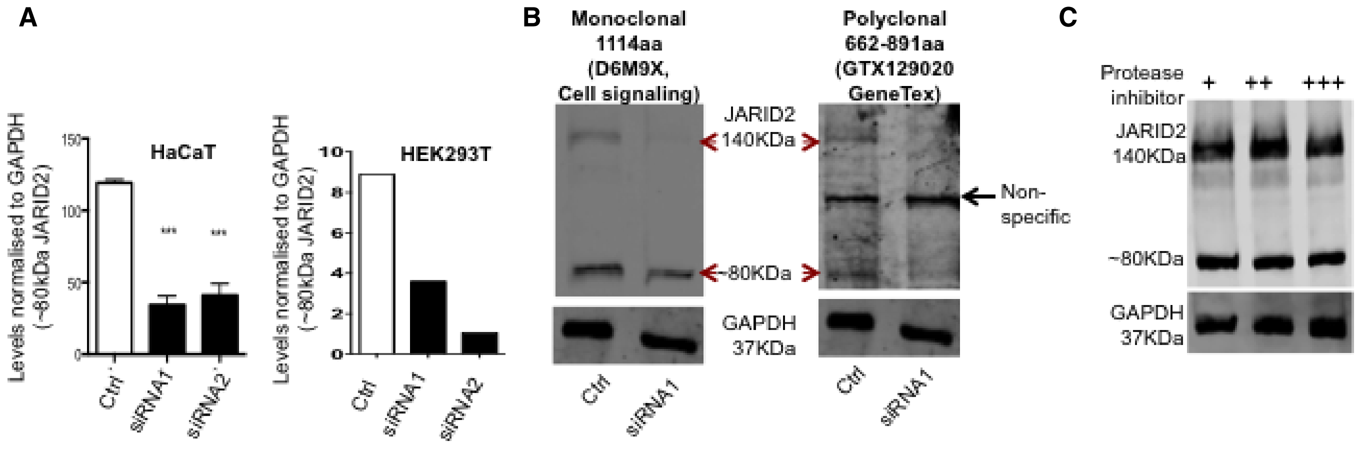

Figure EV1. The 80 kDa band is a JARID2 product.

A Densitometric measurements of the 80kDa band detected in HaCaT and HEK293T cell lysates (corresponding to immunoblots in Fig 1D). Normalised levels of the 80kDa band with respect to GAPDH after transfecting HaCaT and HEK293T cells with non-silencing siRNA control (unfilled bars) and two independent siRNAs (black bars) are shown. Means and SE were calculated based on three independent experiments for HaCaTs. Significance (***$P < 0.001$) was calculated using one-way ANOVA in comparison with non-silencing control. Means were calculated for two independent experiments for HEK293T cells.

B Immunoblots showing the 80kDa band are recognised by two separate JARID2 antibodies. K562 cells transfected with control and JARID2 siRNA1 were blotted with a monoclonal anti-JARID2 antibody (D6M9X, left hand blot), stripped and re-probed with a polyclonal anti-JARID2 antibody (GTX129020, right hand blot). Both antibodies detect the 140 and 80 kDa bands and their reduced expression when transfected with JARID2 siRNA confirms that these are JARID2 bands. The polyclonal antibody is less effective at detecting JARID2 and a non-specific band around 100 kDa was also detected.

C Immunoblot of protein samples from K562 cells extracted in the presence of increasing amounts of protease inhibitor. No change was observed in either the 80 kDa or 140 kDa band confirming that 80 kDa band is not a degradation product.

Source data are available online for this figure.

© 2018 The Authors

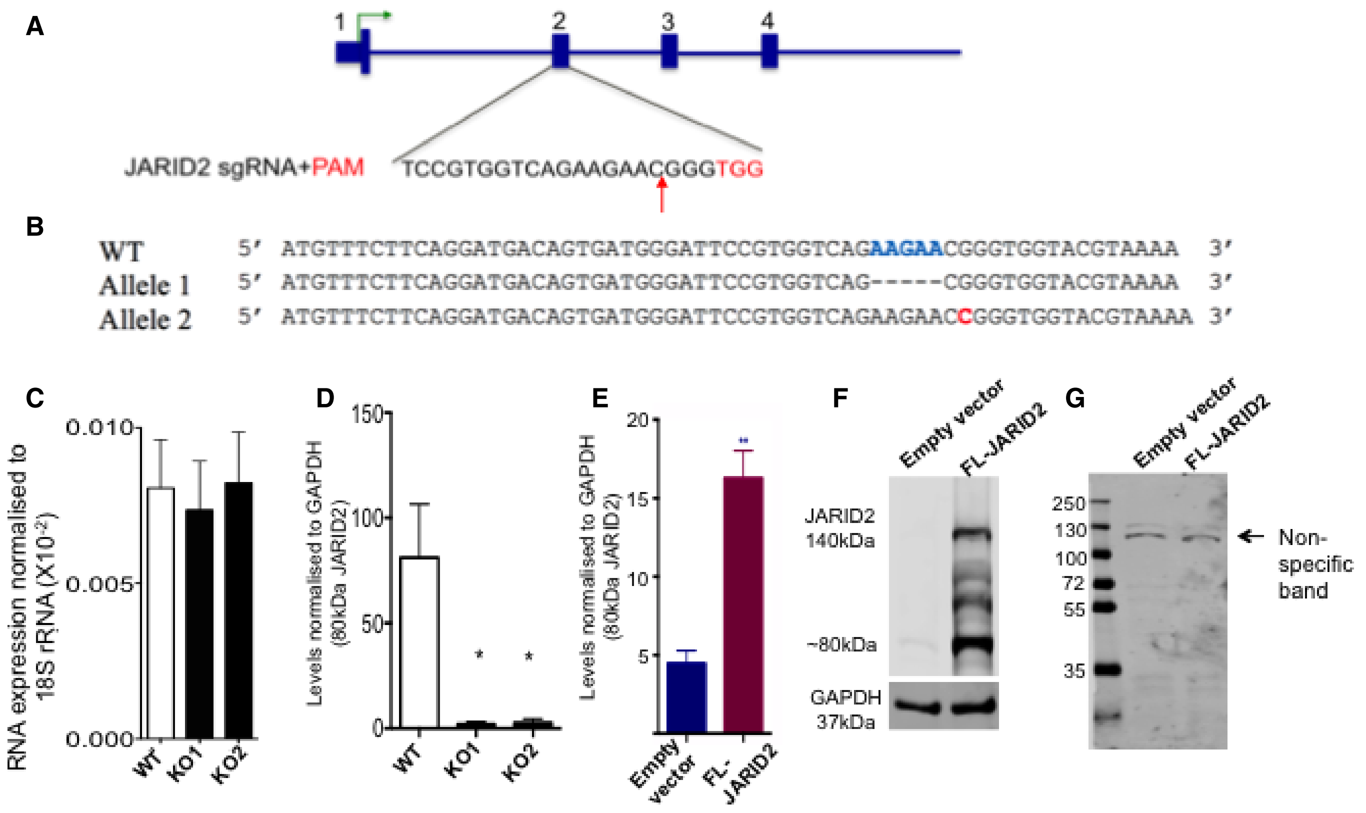

Figure EV2. CRISPR/Cas9-mediated deletion of JARID2.

A Diagram showing location of sgRNA sequence in the JARID2 loci. Red sequence depicts the PAM and red arrow shows the site of double strand cleavage by Cas9 nuclease.

B The DNA sequences of JARID2 targeted alleles show five bases deletion in one copy and a one base insertion in the other copy, resulting in a frameshift in the open reading frame.

C JARID2 mRNA levels measured using qPCR in wild-type (WT) and JARID2 knockout (KO1 and KO2) cells. Data from at least three independent experiments ($n = 3$) are represented as mean ± SE.

D Densitometric measurements (corresponding to immunoblots in Fig 2F) of the 80 kDa band in wild-type (WT) and two JARID2 knockout lines (KO1, KO2) relative to GAPDH levels. Data from three independent experiments ($n = 3$) are represented as mean ± SE, and significance was calculated in multiple comparison using one-way ANOVA (*$P < 0.05$).

E Densitometric measurements (corresponding to immunoblots in Fig 2G) showing an increase in 80kDa band levels relative to GAPDH in cells transfected with full-length JARID2 (FL-JARID2; red) compared to empty vector control (blue). Data from three independent experiments ($n = 3$) are represented as mean ± SE and significance is calculated using *t*-test (**$P < 0.01$).

F Immunoblot of HEK293T cells transfected with FL-JARID2 compared to empty vector control. The blot clearly shows an increase in the levels of the 140 kDa band corresponding to canonical JARID2 indicating expression of the correct protein. The blot also shows an increase in the 80 kDa band.

G Immunoblot with anti-Flag antibody showing that the Flag-tagged N-terminal fragment of FL-JARID2 is not detected in HaCaT cell lysates.

Source data are available online for this figure.

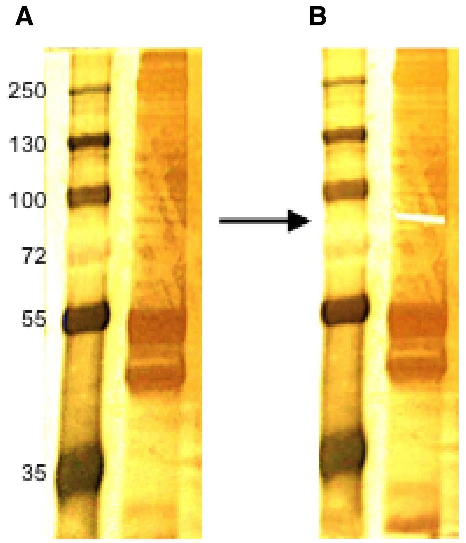

C

	Peptide sequence	Peptide position
1	VIPPPDWRPECK	589-600
2	FVTQIQHIHK	607-616
3	EVGQAQLK	776-783
4	EVVKEEEEDKGVLNDFHK	795-812
5	SVSLTTFYR	819-827
6	VDTNTHGSGFPVGK	868-881
7	LLYQIAQAEAK	1061-1071
8	QLFEAGLHSSAR	1098-1109
9	NGSIENCLSKPTPK	1204-1217
10	ATVDVPPSR	1224-1232

Figure EV3. Mass spectrometry identification of 80kDa band.

A Silver staining of a protein sample electrophoresed on a polyacrylamide gel after immunoprecipitation with JARID2 antibody (D6M9X).
B The ~80 kDa band was cut and subjected to mass spectrometry analysis.
C JARID2 peptides detected in mass spectrometry analysis of ~80 kDa band and their position on JARID2 sequence.

© 2018 The Authors

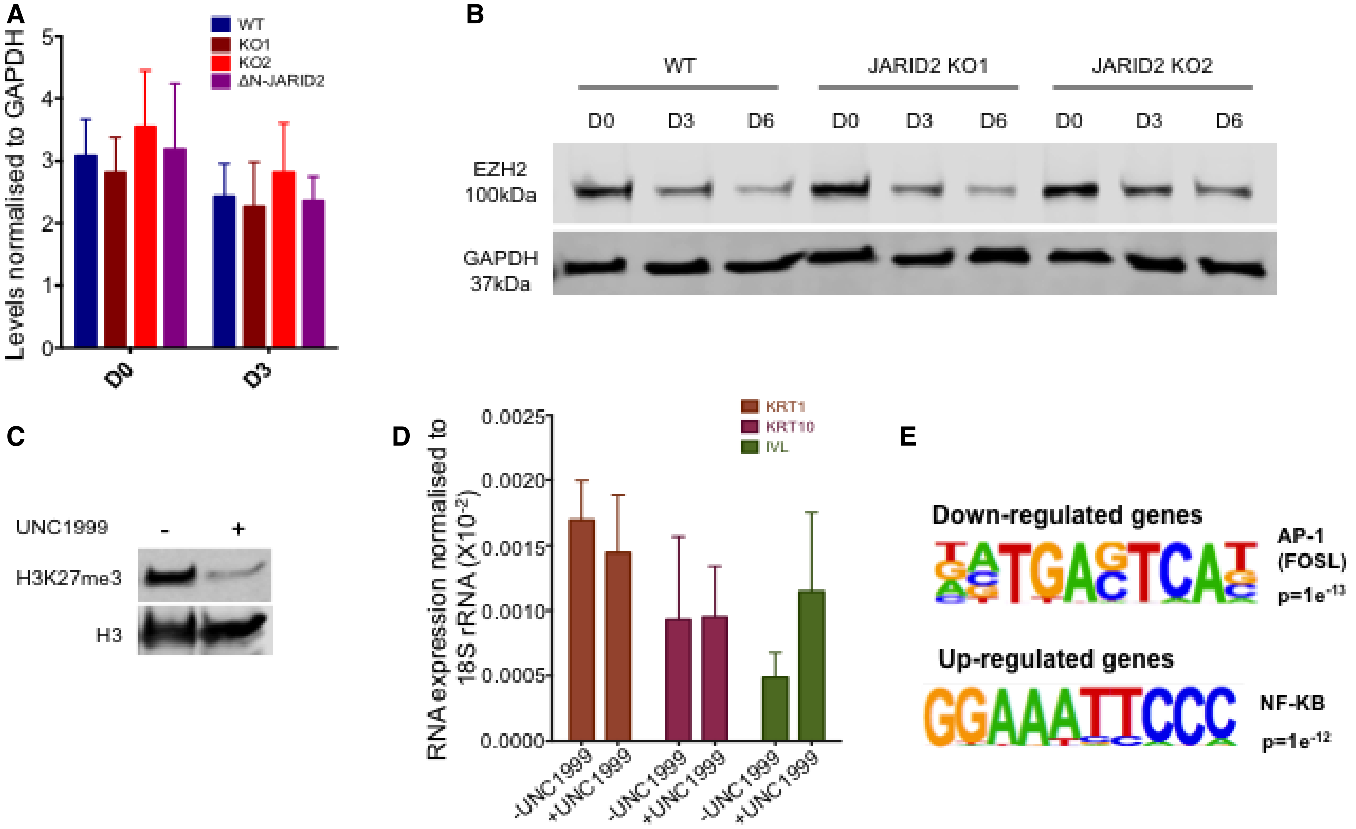

Figure EV4. Relation of JARID2 with H3K27me3 and EZH2 levels.

A Densitometric measurements (*n* = 3) corresponding to Fig 4E, showing there is no change in H3K27me3 between wild-type, JARID2 knockouts and ΔN-JARID2 expressing cells. Data from three independent experiments (*n* = 3) are represented as mean ± SE.

B EZH2 levels in wild type (WT) and two knockout lines of JARID2 (JARID2 KO1 and JARID2 KO2) during day 0, day 3 and day 6 of differentiation. GAPDH is used as loading control.

C H3K27me3 and histone H3 levels in HaCaT cells after 24-h treatment with EZH2 inhibitor UNC1999.

D Bar plots showing expression levels of epidermal differentiation genes before and after treating HaCaT cells with EZH2 inhibitor UNC1999. RNA expression is measured relative to 18S rRNA levels and shows that treatment with EZH2 inhibitor UNC1999 does not lead to significant changes in gene expression of epidermal differentiation genes. Data from three independent experiments (*n* = 3) are represented as mean ± SE.

E Transcription factor motifs in genes affected by JARID2 knockout. The AP-1 motif is the highest enriched motif seen in down-regulated genes, and NF-KB is the highest enriched motif in up-regulated genes.

Source data are available online for this figure.

© 2018 The Authors
