# Supplementary material for: A novel form of JARID2 is required for differentiation in lineage‐committed cells
Source: EMBO J. 2018 Dec 20;38(3):e98449. doi: 10.15252/embj.201798449 (PMC6356158; doi:10.15252/embj.201798449)
Supplement: Supplementary file 5 — Source Data for Figure 1 [file EMBJ-38-e98449-s003.pptx]

## Slide 1
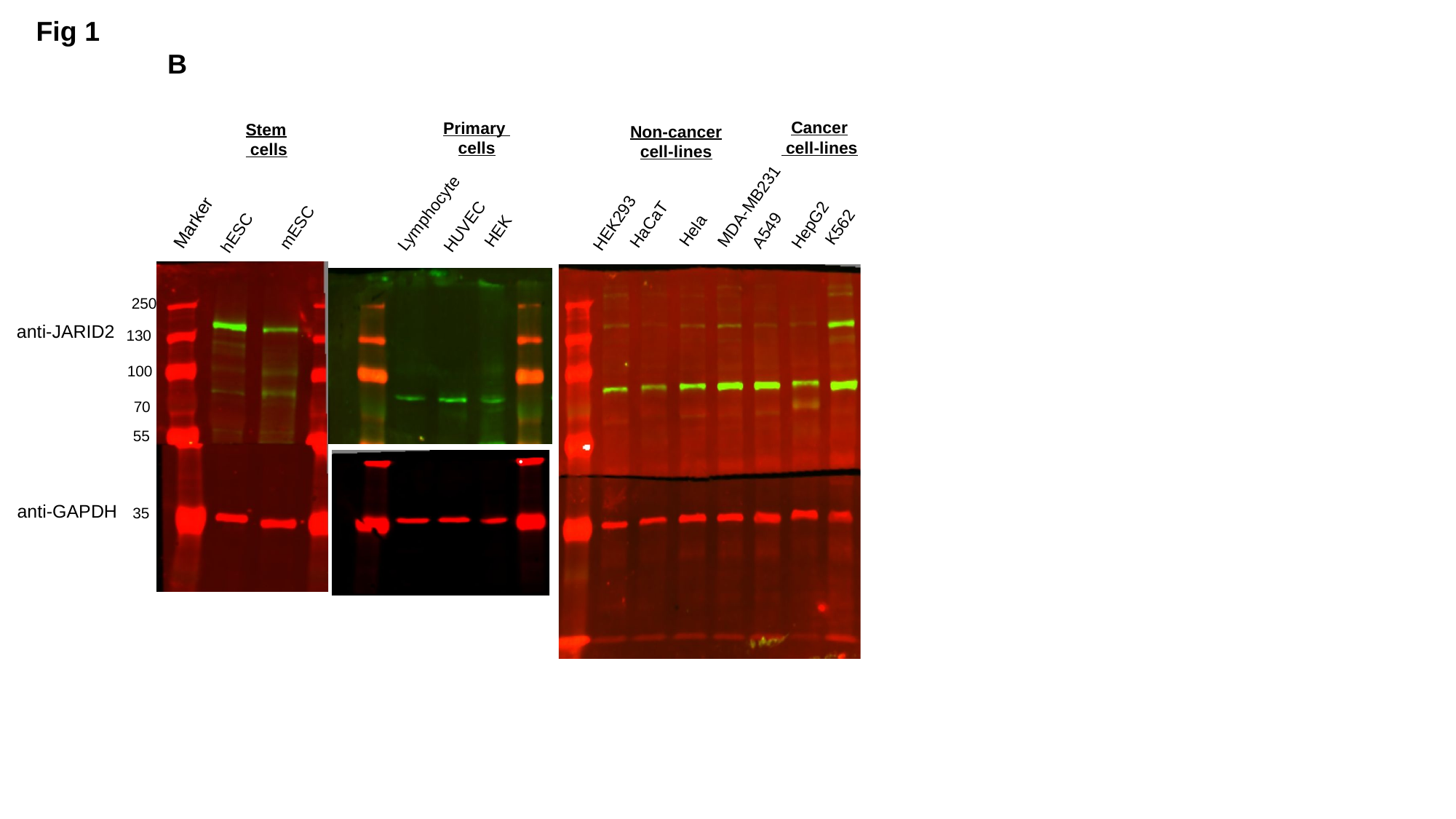

Fig 1
B
Cancer
 cell-lines
Primary
cells
Stem
 cells
Non-cancer
cell-lines
MDA-MB231
A549
HaCaT
HEK293
HepG2
Hela
K562
Marker
mESC
hESC
Lymphocyte
HEK
HUVEC
250
anti-JARID2
130
100
70
55
anti-GAPDH
35

## Slide 2
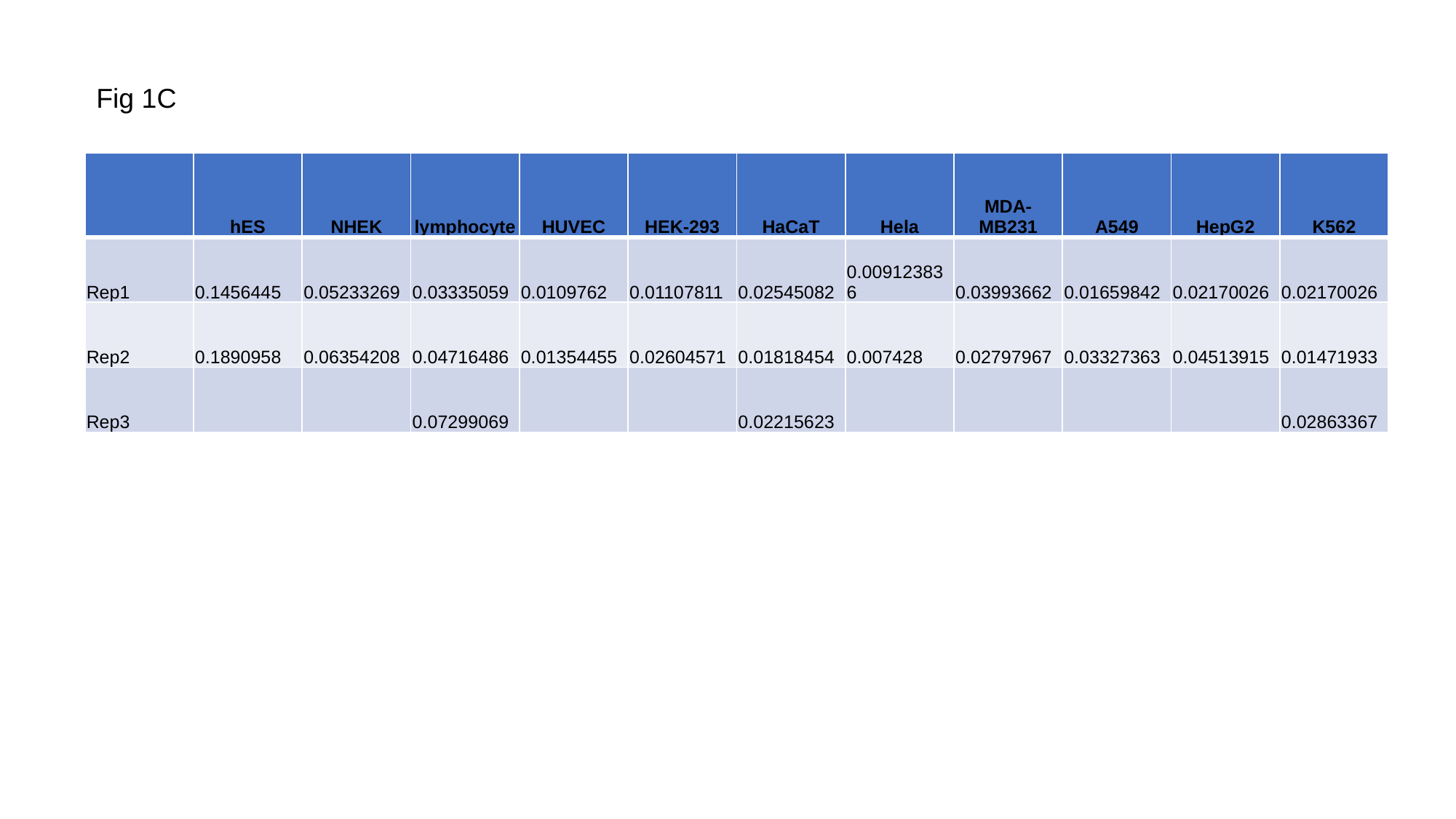

Fig 1C
| | hES | NHEK | lymphocyte | HUVEC | HEK-293 | HaCaT | Hela | MDA-MB231 | A549 | HepG2 | K562 |
| --- | --- | --- | --- | --- | --- | --- | --- | --- | --- | --- | --- |
| Rep1 | 0.1456445 | 0.05233269 | 0.03335059 | 0.0109762 | 0.01107811 | 0.02545082 | 0.009123836 | 0.03993662 | 0.01659842 | 0.02170026 | 0.02170026 |
| Rep2 | 0.1890958 | 0.06354208 | 0.04716486 | 0.01354455 | 0.02604571 | 0.01818454 | 0.007428 | 0.02797967 | 0.03327363 | 0.04513915 | 0.01471933 |
| Rep3 | | | 0.07299069 | | | 0.02215623 | | | | | 0.02863367 |

## Slide 3
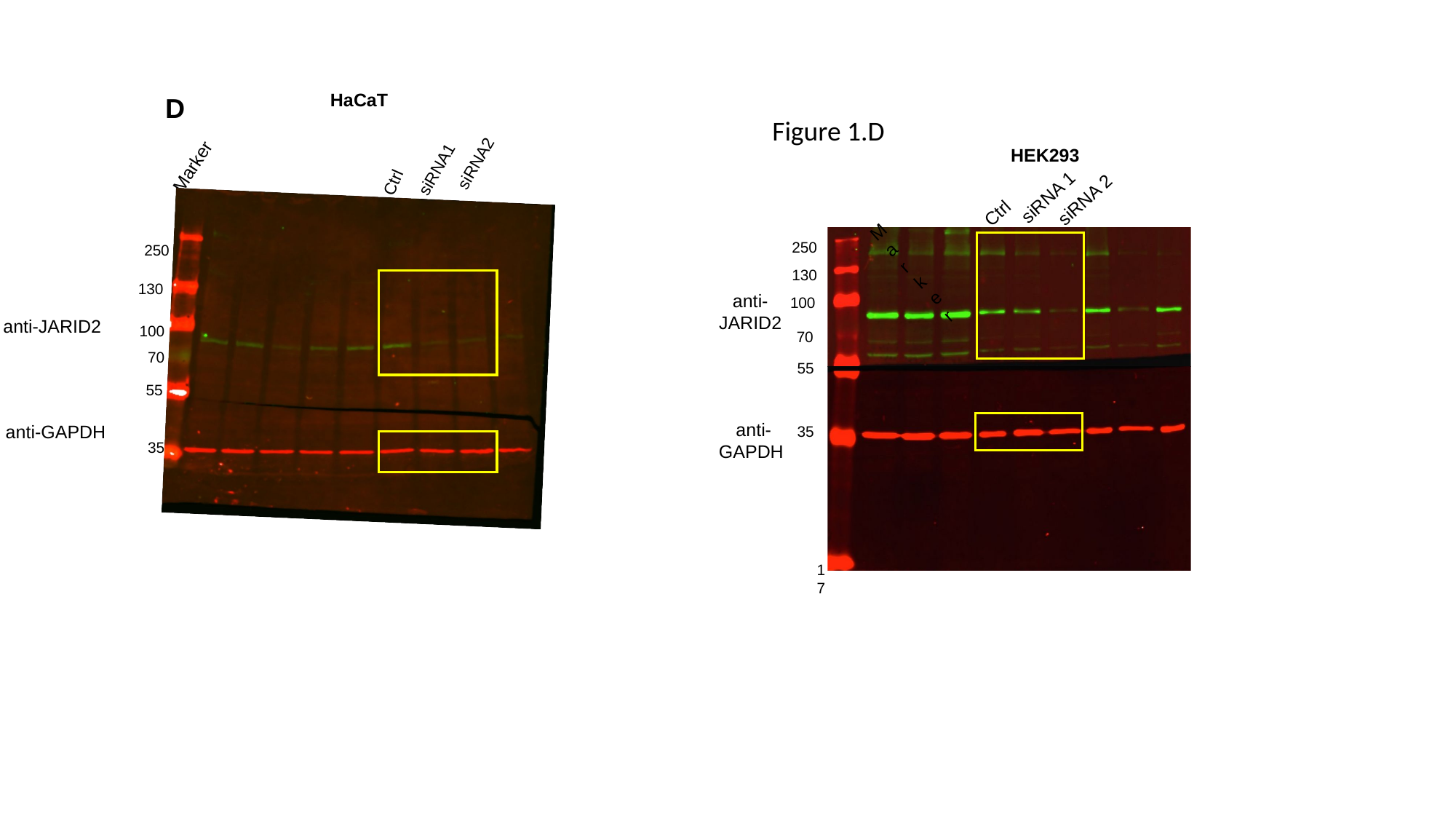

HaCaT
Ctrl
250
130
anti-JARID2
100
70
55
anti-GAPDH
35
siRNA2
siRNA1
Marker
D
Figure 1.D
HEK293
 siRNA 2
 siRNA 1
 Marker
 Ctrl
250
130
anti-JARID2
100
70
55
anti-GAPDH
17
35
