# Supplementary material for: A novel form of JARID2 is required for differentiation in lineage‐committed cells
Source: EMBO J. 2018 Dec 20;38(3):e98449. doi: 10.15252/embj.201798449 (PMC6356158; doi:10.15252/embj.201798449)
Supplement: Supplementary file 6 — Source Data for Figure 2 [file EMBJ-38-e98449-s004.pptx]

## Slide 1
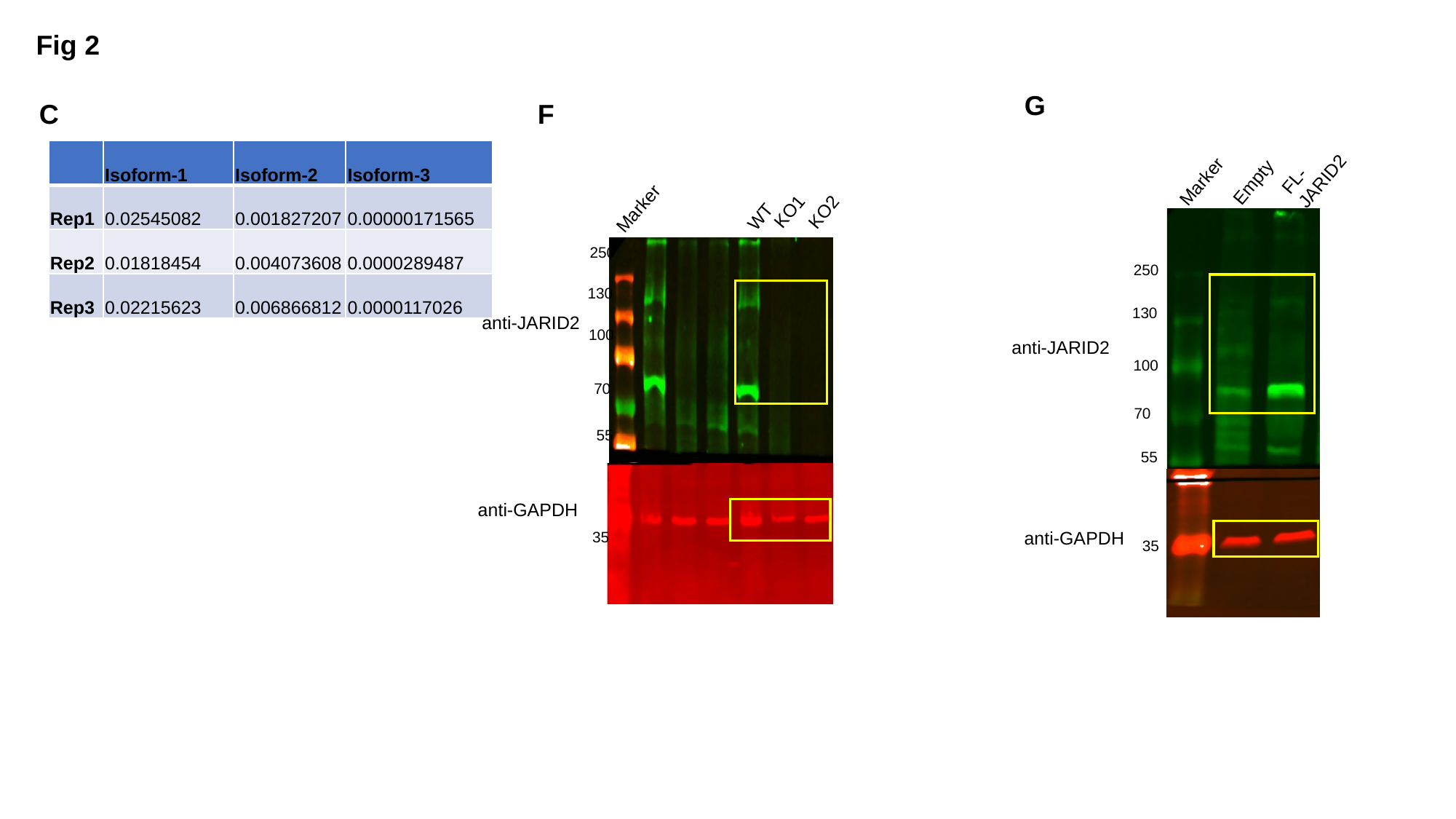

Fig 2
 FL-
 JARID2
 Empty
Marker
250
130
100
70
55
35
anti-JARID2
anti-GAPDH
G
C
F
WT
KO1
KO2
250
130
anti-JARID2
100
70
55
anti-GAPDH
35
| | Isoform-1 | Isoform-2 | Isoform-3 |
| --- | --- | --- | --- |
| Rep1 | 0.02545082 | 0.001827207 | 0.00000171565 |
| Rep2 | 0.01818454 | 0.004073608 | 0.0000289487 |
| Rep3 | 0.02215623 | 0.006866812 | 0.0000117026 |
Marker
