# Supplementary material for: A novel form of JARID2 is required for differentiation in lineage‐committed cells
Source: EMBO J. 2018 Dec 20;38(3):e98449. doi: 10.15252/embj.201798449 (PMC6356158; doi:10.15252/embj.201798449)
Supplement: Supplementary file 7 — Source Data for Figure 3 [file EMBJ-38-e98449-s005.pptx]

## Slide 1
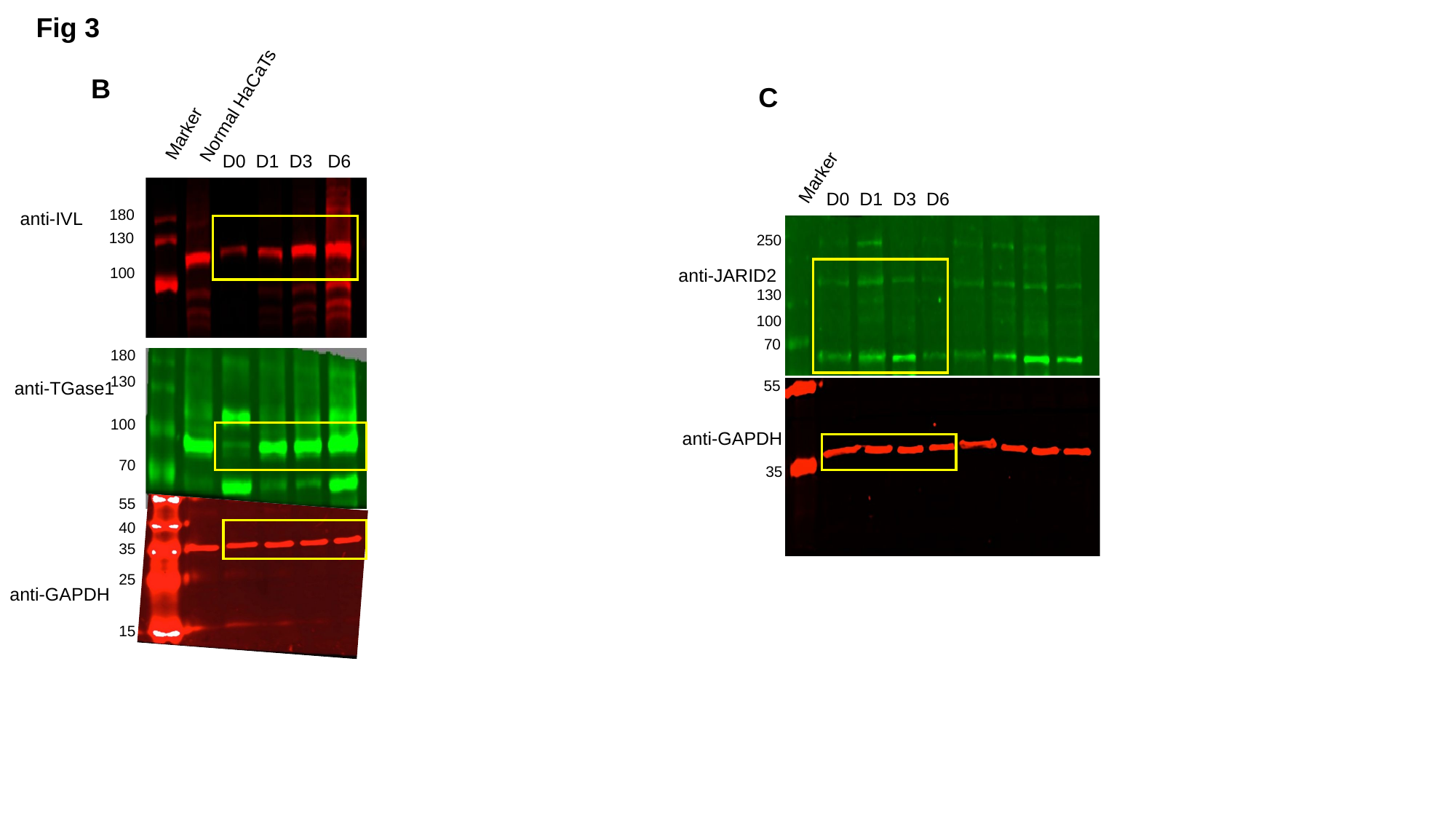

Fig 3
B
C
 D0 D1 D3 D6
250
anti-JARID2
130
100
70
55
anti-GAPDH
35
Marker
 Normal HaCaTs
Marker
 D0 D1 D3 D6
180
anti-IVL
130
100
180
130
anti-TGase1
100
70
55
40
35
25
anti-GAPDH
15

## Slide 2
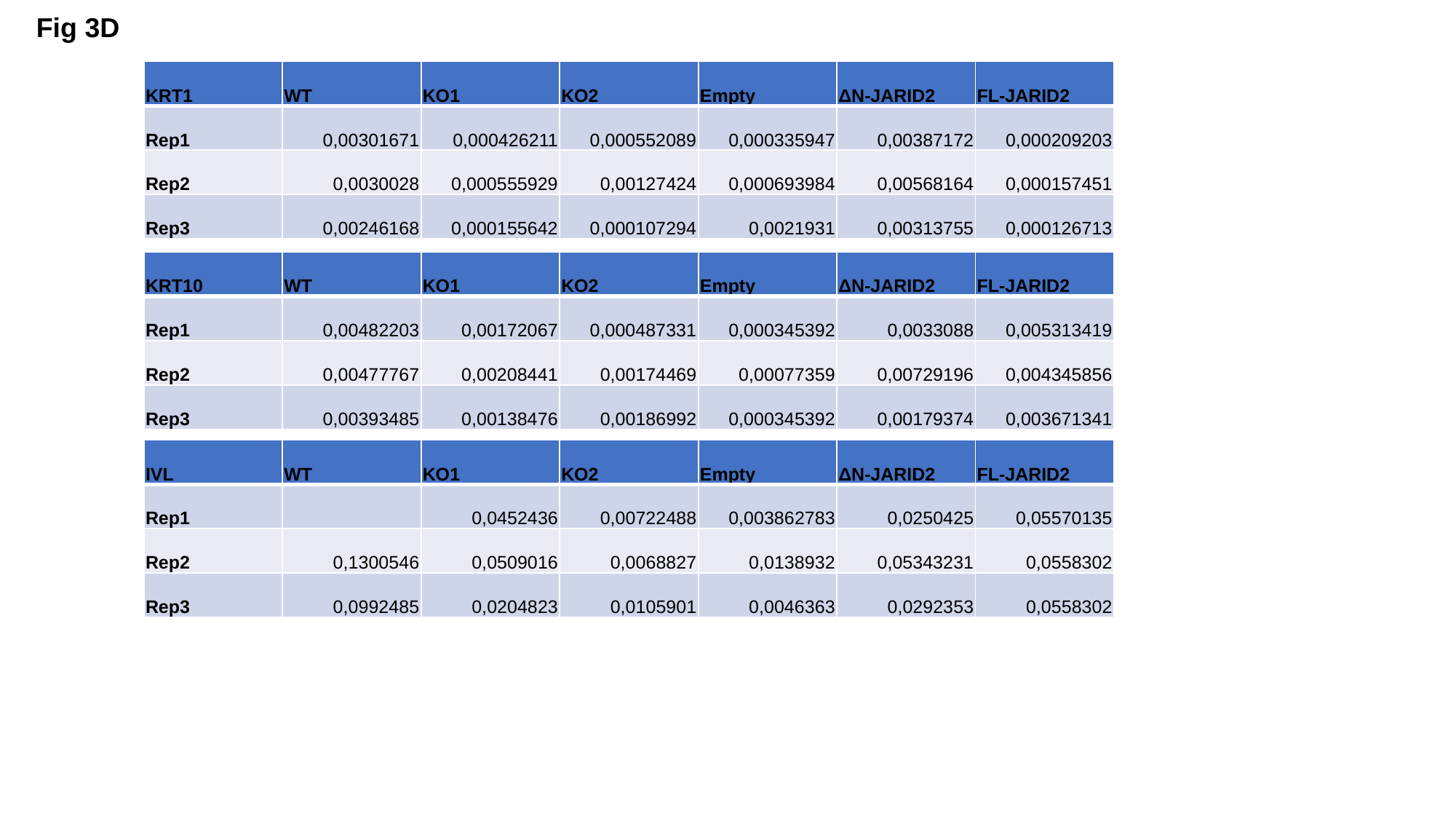

Fig 3D
| KRT1 | WT | KO1 | KO2 | Empty | ΔN-JARID2 | FL-JARID2 |
| --- | --- | --- | --- | --- | --- | --- |
| Rep1 | 0,00301671 | 0,000426211 | 0,000552089 | 0,000335947 | 0,00387172 | 0,000209203 |
| Rep2 | 0,0030028 | 0,000555929 | 0,00127424 | 0,000693984 | 0,00568164 | 0,000157451 |
| Rep3 | 0,00246168 | 0,000155642 | 0,000107294 | 0,0021931 | 0,00313755 | 0,000126713 |
| KRT10 | WT | KO1 | KO2 | Empty | ΔN-JARID2 | FL-JARID2 |
| --- | --- | --- | --- | --- | --- | --- |
| Rep1 | 0,00482203 | 0,00172067 | 0,000487331 | 0,000345392 | 0,0033088 | 0,005313419 |
| Rep2 | 0,00477767 | 0,00208441 | 0,00174469 | 0,00077359 | 0,00729196 | 0,004345856 |
| Rep3 | 0,00393485 | 0,00138476 | 0,00186992 | 0,000345392 | 0,00179374 | 0,003671341 |
| IVL | WT | KO1 | KO2 | Empty | ΔN-JARID2 | FL-JARID2 |
| --- | --- | --- | --- | --- | --- | --- |
| Rep1 | | 0,0452436 | 0,00722488 | 0,003862783 | 0,0250425 | 0,05570135 |
| Rep2 | 0,1300546 | 0,0509016 | 0,0068827 | 0,0138932 | 0,05343231 | 0,0558302 |
| Rep3 | 0,0992485 | 0,0204823 | 0,0105901 | 0,0046363 | 0,0292353 | 0,0558302 |

## Slide 3
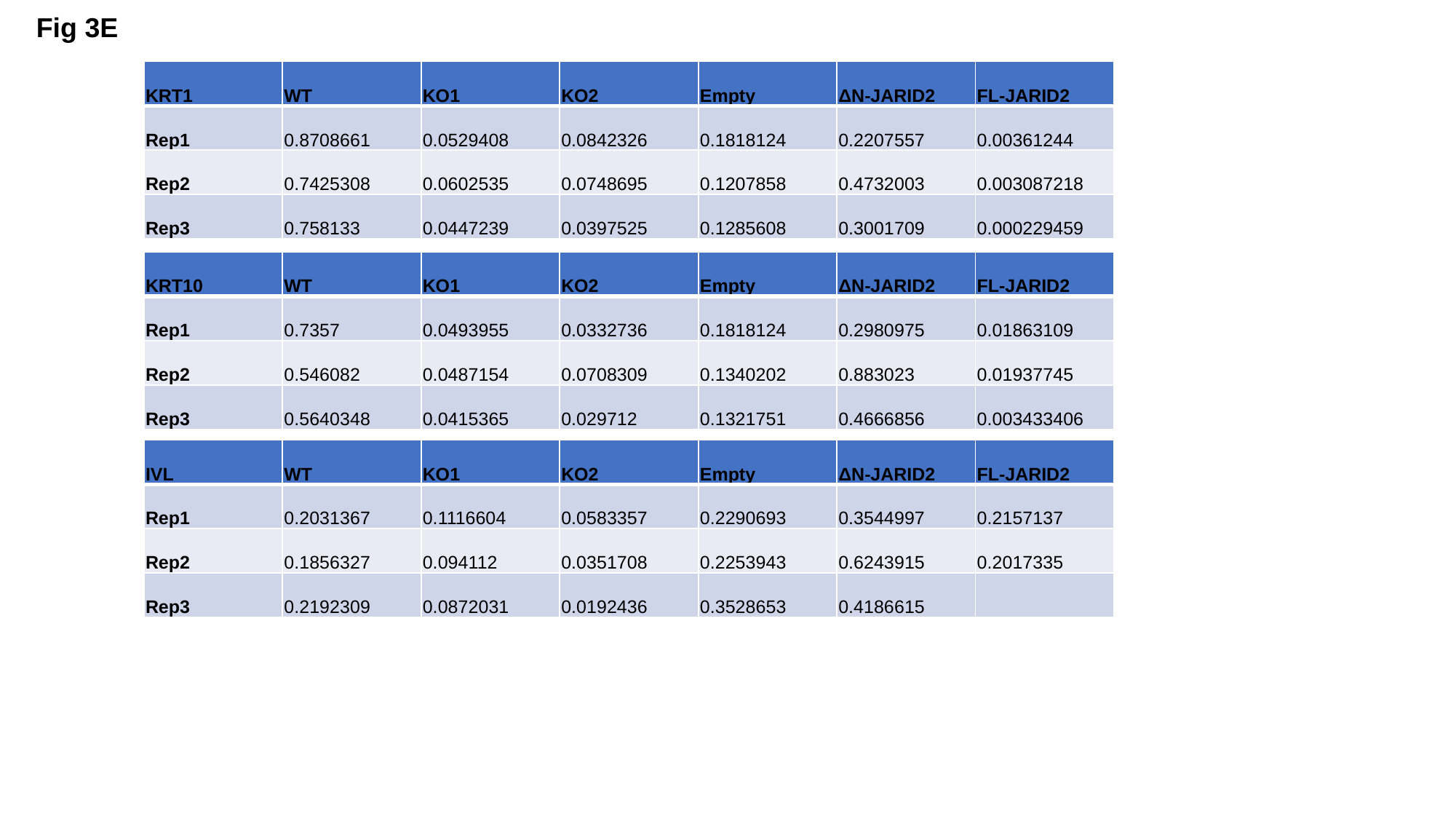

Fig 3E
| KRT1 | WT | KO1 | KO2 | Empty | ΔN-JARID2 | FL-JARID2 |
| --- | --- | --- | --- | --- | --- | --- |
| Rep1 | 0.8708661 | 0.0529408 | 0.0842326 | 0.1818124 | 0.2207557 | 0.00361244 |
| Rep2 | 0.7425308 | 0.0602535 | 0.0748695 | 0.1207858 | 0.4732003 | 0.003087218 |
| Rep3 | 0.758133 | 0.0447239 | 0.0397525 | 0.1285608 | 0.3001709 | 0.000229459 |
| KRT10 | WT | KO1 | KO2 | Empty | ΔN-JARID2 | FL-JARID2 |
| --- | --- | --- | --- | --- | --- | --- |
| Rep1 | 0.7357 | 0.0493955 | 0.0332736 | 0.1818124 | 0.2980975 | 0.01863109 |
| Rep2 | 0.546082 | 0.0487154 | 0.0708309 | 0.1340202 | 0.883023 | 0.01937745 |
| Rep3 | 0.5640348 | 0.0415365 | 0.029712 | 0.1321751 | 0.4666856 | 0.003433406 |
| IVL | WT | KO1 | KO2 | Empty | ΔN-JARID2 | FL-JARID2 |
| --- | --- | --- | --- | --- | --- | --- |
| Rep1 | 0.2031367 | 0.1116604 | 0.0583357 | 0.2290693 | 0.3544997 | 0.2157137 |
| Rep2 | 0.1856327 | 0.094112 | 0.0351708 | 0.2253943 | 0.6243915 | 0.2017335 |
| Rep3 | 0.2192309 | 0.0872031 | 0.0192436 | 0.3528653 | 0.4186615 | |
