# Supplementary figures and images for: A novel form of JARID2 is required for differentiation in lineage‐committed cells
Source: EMBO J. 2018 Dec 20;38(3):e98449. doi: 10.15252/embj.201798449 (PMC6356158; doi:10.15252/embj.201798449)

## Slide 1
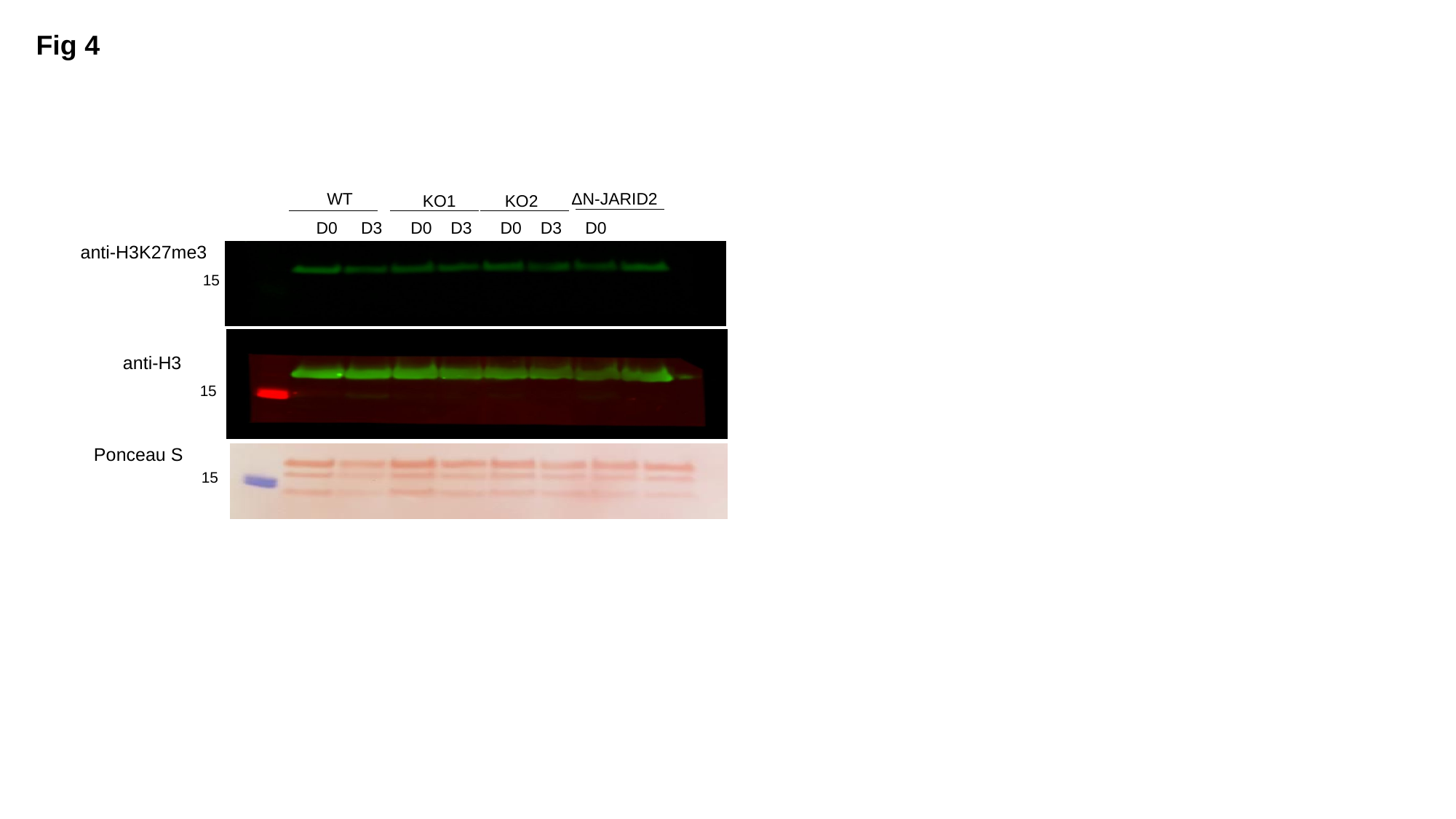

Fig 4
WT
ΔN-JARID2
KO1
KO2
 D0 D3 D0 D3 D0 D3 D0 D3
anti-H3K27me3
15
anti-H3
15
Ponceau S
15

Supplement: Supplementary file 8 — Source Data for Figure 4 [file EMBJ-38-e98449-s006.pptx]
